# Harnessing T Follicular Helper Cell Responses for HIV Vaccine Development

**DOI:** 10.3390/v10060336

**Published:** 2018-06-19

**Authors:** Julia Niessl, Daniel E. Kaufmann

**Affiliations:** 1 Centre de Recherche du Centre Hospitalier de l’Université de Montréal (CRCHUM) and University of Montreal, Montreal, QC H2X 0A9, Canada; julia.niessl@umontreal.ca; 2 Scripps Center for HIV/AIDS Vaccine Immunology and Immunogen Discovery (CHAVI-ID), La Jolla, CA 92037, USA

**Keywords:** CD4 T cell help, T follicular helper cells (Tfh), B cells, antibody, broadly neutralizing antibody (bNAb), HIV, vaccine

## Abstract

Passive administration of broadly neutralizing antibodies (bNAbs) capable of recognizing a broad range of viral strains to non-human primates has led to protection from infection with chimeric SIV/HIV virus (SHIV). This data suggests that generating protective antibody responses could be an effective strategy for an HIV vaccine. However, classic vaccine approaches have failed so far to induce such protective antibodies in HIV vaccine trials. HIV-specific bNAbs identified in natural infection show high levels of somatic hypermutations, demonstrating that they underwent extensive affinity maturation. It is likely that to gain ability to recognize diverse viral strains, vaccine-induced humoral responses will also require complex, iterative maturation. T follicular helper cells (Tfh) are a specialized CD4+ T cell subset that provides help to B cells in the germinal center for the generation of high-affinity and long-lasting humoral responses. It is therefore probable that the quality and quantity of Tfh responses upon vaccination will impact development of bNAbs. Here, we review studies that advanced our understanding of Tfh differentiation, function and regulation. We discuss correlates of Tfh responses and bNAb development in natural HIV infection. Finally, we highlight recent strategies to optimize Tfh responses upon vaccination and their impact on prophylactic HIV vaccine research.

## 1. Introduction

Most successful vaccines (e.g., against hepatitis B, yellow fever, and smallpox) work by inducing long-lasting neutralizing antibody responses that prevent infection of target cells [1]. Current human immunodeficiency virus (HIV) prevention strategies, including public awareness campaigns, condom use, and post-exposure prophylaxis, led to a decline of the annual number of new HIV infections to 1.8 million worldwide. In addition, full suppression of viral replication by antiretroviral therapy (ART) in HIV-infected individuals strongly reduces transmission rates. However, ending the pandemic without an effective vaccine seems unlikely [2].

Env is the only viral protein expressed on the surface of free, mature HIV virions. Broadly neutralizing antibodies (bNAbs) are able to recognize a variety of different HIV strains by targeting conserved regions of the HIV envelope protein. Passive administration of bNAbs has been shown to prevent infection in non-human primate (NHP) models [3,4,5]. In these studies, infused animals were challenged with Simian Human Immunodeficiency Virus (SHIV), a chimeric viral construct with an HIV envelope in an SIV backbone, which allows studying humoral responses against HIV in an animal model. These results suggest that vaccine-induced protective antibody responses could also serve as a strategy for an HIV vaccine. However, the induction of long-lasting bNAbs responses remains a major challenge and has been unsuccessful in human HIV vaccine trials [6].

High quality and long-lived humoral immune responses require help from a specialized CD4+ T cell subset called T follicular helper cells (Tfh) [7]. Tfh cells differentiate from naïve CD4+ T cells upon interaction with antigen-presenting dendritic cells (DCs) and migrate to the germinal center (GC) of secondary lymphoid organs. There, they control B cell proliferation, affinity maturation, class-switch recombination (CSR), and long-lasting memory formation. They therefore play an important role in the generation of protective antibody responses [7]. HIV is characterized by exceptionally high mutation rates, and the human immune system lags behind the evolution of autologous strains: most circulating viruses are resistant to neutralizing antibodies in serum from the same time point. After years of infection, a minority of HIV individuals (in the range of 10–20%) develop potent antibodies capable of neutralizing diverse primary isolates [8]. In contrast to neutralizing antibodies against most other pathogens, these potent HIV-specific bNAbs usually exhibit high rate of somatic hypermutations (SHM), which are necessary for neutralizing potency and breath. This suggests that HIV-bNAbs must have undergone multiple rounds of affinity maturation in the GC [9]. It is therefore likely that, compared to conventional vaccine strategies, more efficient Tfh responses are required for the generation of HIV-specific bNAbs. In this review, we highlight recent findings on Tfh differentiation, function and regulation as well as correlates of Tfh responses and bNAb development during natural HIV infection. We report on strategies to optimize Tfh and GC responses for the induction of effective antibody responses, some of which have already shown some success in non-HIV-vaccines in humans or HIV-related vaccine studies in NHPs. These findings may guide future approaches for the development of a prophylactic HIV vaccine.

## 2. Tfh Differentiation

Tfh cells are a specialized CD4+ T helper subset, characterized by the expression of CXCR5, the ligand for the chemokine CXCL13, which allows their migration into the GC of secondary lymphoid organs [10,11]. There, they provide B cell help for the generation of high affinity antibody responses. Further phenotypic and functional markers include Bcl6, PD-1, ICOS, CD40L and IL-21, which are important for differentiation and function of Tfh cells and can be expressed at different levels depending on the differentiation status.

**Figure 1:**
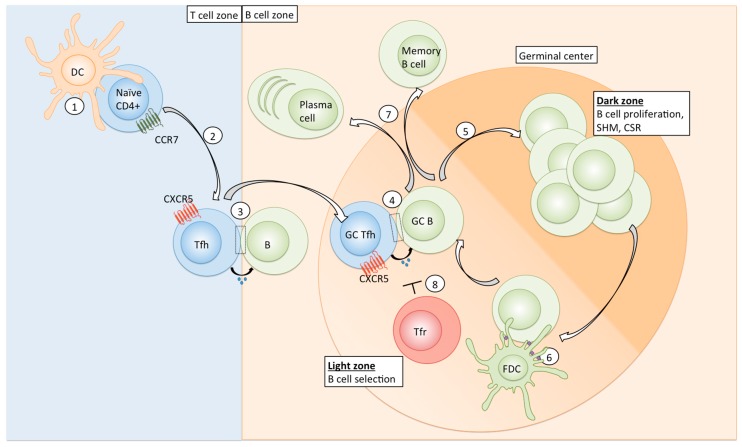
Overview of Tfh differentiation and function. Naïve CD4+ T cells are primed by antigen-presenting dendritic cells in the T cell zone of secondary lymphoid organs (1); Specific cytokines, co-signalling surface receptor molecules (see Table 1) and a prolonged DC-naïve CD4 interaction favour Tfh commitment. Tfh-polarized cells downregulate CCR7 and express CXCR5, the CXCL13 ligand, which allows their migration to the T-B-border (2); where first interaction with B cells occurs (3); Tfh cells then migrate into the germinal center, where further interaction with GC B cells sustains the GC Tfh polarization (4); In the dark zone of the GC, B cells undergo proliferation, affinity maturation via SHM, and CSR (5); B cells migrate to the light zone to receive survival and selection signals. They take up and process antigen (purple) from FDCs (6) and subsequently present it to GC Tfh cells (4). High affinity B cells are able to capture and present more antigen and therefore receive more Tfh cell help. Repeated circulation of B cells between DZ and LZ results in the acquisition of high levels of SHM and selection of high affinity clones. B cells eventually differentiate into antibody-producing plasma cells or memory B cells and enter the blood circulation (7); Tfr cells can inhibit GC Tfh and B cell responses via multiple mechanisms (8); DC: dendritic cell, Tfh: T follicular helper cell, SHM: somatic hypermutation, CSR: class switch recombination, FDC: follicular dendritic cell, DZ: dark zone; LZ: light zone; Tfr: follicular T regulatory cell.

Tfh differentiation is a multifactorial and multistep process (see Overview, Figure 1). Initially, naïve CD4+ T cells are primed by antigen-presenting DCs in the T cell zone of secondary lymphoid organs. Early expression of the transcription factors Lef-1 and Tcf-1 primes naïve CD4+ T cells for further Tfh-promoting signals and leads to the upregulation of the transcriptional repressor Bcl6 [12,13], which is absolutely required for Tfh development [14,15,16]. Bcl6 acts together with other Tfh-related transcription factors (e.g., Maf and Ascl2) to repress non-Tfh related signature genes and induce key Tfh-associated genes such as PD-1 and CXCR5 [14,17,18]. Expression of CXCR5 and concomitant downregulation of CCR7 on the cell surface allows early Tfh cells to migrate to the T-B border [19]. There, Tfh cells interact with antigen-presenting B cells via ICOS-ICOSL [20], which leads to the reinforcement and persistence of the Tfh signature, and migration into the B cell follicle for the formation of GCs [21]. Further interactions between Tfh and antigen-presenting B cells in the GC are necessary to sustain Tfh commitment, demonstrating that continuous antigenic activation is important for their maintenance [22].

Signalling molecules involved in the positive or negative regulation of Tfh differentiation present some notable differences between mice and humans and are shown in Table 1. In addition, quantitative signals related to strong and prolonged interaction between the T cell receptor and the major histocompatibility complex class II (MHCII) molecule favours Tfh vs. non-Tfh commitment [23]. A better understanding of how Tfh differentiation can be regulated in humans is of great importance as these pathways might serve as target to induce and regulate Tfh responses for vaccine strategies.

## 3. T-Cell Dependent Antibody Responses

B cell differentiation and isotype switch can occur after initial T-B interaction and outside of the GC. This extrafollicular response emerges early after immunization and provides a first line of protective antibodies upon infection [51]. However, plasma cells generated following this type of interaction are usually short-lived and of low affinity due to only minimal affinity maturation [51]. For the efficient neutralization of viruses and other pathogens, high affinity antibodies are required. In addition, induction of long-lived memory B cell responses after infection or vaccination is desired to ensure long-term immunity. Both can be achieved in the GC reaction.

GC Tfh cells play a central role as they regulate multiple aspects of this process: B cell survival, proliferation, SHM, CSR, and differentiation. Tfh cells reside in the light zone of a mature GC. There, B cells take up antigen from follicular dendritic cells (FDCs), process it and present it to GC Tfh via MHCII. During this process, B cells compete for limited Tfh help: High affinity B cells, which were able to capture and therefore present more antigen compared to B cells with lower affinity, are more likely to receive Tfh signals [52,53]. Selected B cells enter the dark zone (DZ), where they proliferate and undergo SHM of the B cell receptor (BCR) V-region genes, the rate of which directly correlates with the Tfh help received in the light zone (LZ) [54]. During this process, mainly single nucleotide exchanges are introduced, resulting in a random modification of the BCR binding-affinity. GC B cell clones return to the LZ and are further selected by Tfh based on their antigen binding capacity. Repeated circulation between the LZ for selection for high-affinity and DZ for proliferation and affinity maturation results in the acquisition of elevated rates of somatic mutations and ensures the dominance of high-affinity B cell clones. Eventually, B cells differentiate into long-lived plasma cells or memory B cells and enter the circulation, thus allowing seeding of other anatomic locations.

Tfh help in the GC occurs via direct cell-cell interactions with B cells and secretion of cytokines. Some important mediators for B cell survival, proliferation, and differentiation are summarized in Table 2. In addition, B cell functions and differentiation can be complemented and modulated by a variety of other cytokines that regulate alone or in combination CSR and differentiation and thus outcome of antibody responses (reviewed in [55]). Signals regulating GC B cell differentiation to plasma cell vs. memory B cells are not well understood but recent studies suggested that high affinity B cell antigen interaction and IL-21 produced by Tfh cells favour plasma cell differentiation [56,57].

## 4. Memory and Circulating Tfh Cells

GC Tfh cells have been shown to form a pool of memory cells upon antigen clearance in both mice and humans. Memory Tfh cells localize together with antigen-specific memory B cells in the draining lymph node (LN) for the rapid induction of humoral responses upon re-exposure to antigen [65]. A subset of CD4 T cells in peripheral blood, termed circulating Tfh (cTfh) or peripheral Tfh (pTfh), shares several features with tissue Tfh [66]. cTfh cells have a memory phenotype and express CXCR5, although at lower levels compared to their GC counterparts [67]. In addition, certain phenotypic markers of GC Tfh, e.g., BCL6, are lost or downregulated [68]. Studies in mice showed that cTfh cells originate from GC Tfh cells that left the GC into the blood. Upon activation cTfh cells can migrate to the GC secondary lymphoid organs for the interaction with B cells [69]. In humans, matched samples from blood and tonsils revealed that after vaccination clonal relatives of GC Tfh enter the circulation [70]. Despite the phenotypic differences, functional properties of cTfh cells are partially preserved when compared to their tissue counterparts: cTfh cells express higher levels of Tfh-related cytokines such as IL-21 and CXCL13 and show a superior capacity for B cell help in in vitro co-culture assays when compared to CXCR5- non-cTfh cells [66]. Of note, all cells identified by a given set of markers as cTfh in blood may not have the same potential to home to lymphoid tissue and become activated GC Tfh. While a better understanding of the relationships between quantitative and qualitative characteristics of cTfh responses and GC activity is thus necessary, monitoring of cTfh and antigen-specific cTfh responses in blood can represent an alternative investigational tool during infection or in vaccine trials when access to lymphoid tissue is limited or not possible.

cTfh represent a heterogeneous population that can be classified into multiple subsets based on polarization and activation status. Differential expression of the chemokine receptors CXCR3 and CCR6 allows the distinction of Th1-like (CXCR3+CCR6-), Th1Th17-like (CXCR3+CCR6+), Th17-like (CXCR3−CCR6+), and Th2-like (CXCR3−CCR6-) cTfh subsets. These cTfh subsets express transcription factors and can produce cytokines upon stimulation that are typically associated with Th1, Th2, Th17, and Th1Th17 CD4+ subsets [66]. Using these surface markers, several groups have identified a differential B cell helper capacity of cTfh subsets in in vitro culture assays: CXCR3- populations were able to provide help for naïve and memory B cells and induced proliferation, differentiation, and class-switched antibody production after stimulation with SEB [66,67,71]. In contrast, CXCR3+ cTfh cells were able to provide help to memory B cells in vitro [71], suggesting a role in promoting recall responses instead of priming primary antibody responses.

Tfh subsets based on the expression of CXCR3 and CCR6 can also be identified in the LN of macaques [72]. However, it remains to be determined whether the helper potential of different cTfh subsets can be translated into GC Tfh cells in tissues.

## 5. Regulation of GC Tfh Responses

Given the important role of Tfh cells for the generation of high-affinity antibody responses, it is not surprising that absence of or impaired Tfh responses hamper the generation of protective antibodies after infection or vaccination [73]. However, on the other hand, excessive accumulation of an overactive GC Tfh response correlated with the development of antibody-mediated autoimmunity or the generation of low affinity antibody responses by allowing the survival of B cells with self-reactivity or low binding capacity [74,75]. This shows that Tfh number and function in the GC needs to be regulated to ensure an efficient and targeted B cell help. Indeed, Tfh cells are only a minor population in the GC to allow competition of B cells for limited help and selection of only high-affinity clones. Tfh number can be regulated at the stage of differentiation as mentioned above. In addition, several mechanisms control Tfh number and function in the GC.

Tfh cells are characterized by the high expression of multiple co-inhibitory receptors including PD-1, TIGIT or CD200 [67,76,77]. While PD-1 and likely other of these molecules are required for interaction with B cells and additional cell types to ensure proper humoral responses [78], they might also be involved in the regulation of Tfh expansion and function in the context of chronic antigen exposure in the GC environment. For example, knockout or blocking of the immune checkpoints CTLA-4 or PD-1, alone or in combination with Lag-3, induced Tfh proliferation and enhanced cytokine production [50,79,80].

In addition, GC responses are controlled by the recently identified T follicular regulatory cells (Tfr) [81,82,83]. Tfr cells express similar phenotypic markers compared to Tfh cells including CXCR5, PD-1, Bcl6, and ICOS [77,81,82,83]. In contrast to Tfh cells, Tfr cells differentiate from natural Tregs and express Foxp3 and Helios [83]. The mechanism of Tfr-mediated GC regulation is-especially in humans-not well understood. Studies in mice demonstrated that Tfr cells inhibit proliferation and cytokine expression in Tfh cells as well as CSR and antibody production in B cells [69,84]. These effects were mediated via Tfr-induced changes in the cellular metabolism of Tfh and B cells that were long lasting but reversible and partially due to epigenetic modifications [84]. Additional Tfr-mediated suppressor mechanisms may include the physical inhibition of Tfh-B-interaction, induction of cell death via granzyme B, and the expression of inhibitory cytokines (reviewed in [85]). Negative regulators of Tfh cell number and function might serve as additional target for the induction of high-affinity antibody responses during infection or vaccination.

## 6. Tfh Cells during HIV Infection and Correlates with bNAb Development

Tfh cell are highly permissive to HIV or SIV infection [86,87,88], yet GC Tfh cell number and frequency is increased in infected animals and subjects [86,87,89]. This is driven by clonal expansion of chronically stimulated HIV-specific GC Tfh cells [90], and a Tfh-favourable cytokine milieu that induces general non-HIV-specific Tfh differentiation. Virus-specific Tfh expansion occurs in the context of other chronic viral infections such as hepatitis C in humans or lymphocytic choriomeningitis in mice [91,92]. In addition to their accumulation, GC Tfh cells showed phenotypic and functional changes during HIV or SIV infection that correlated with dysregulated B cell responses: GC Tfh expansion was associated with an elevated number of GC B cells and plasma cells, accompanied by hypergammaglobulinaemia, whereas memory B cells were decreased in chronically infected individuals [89]. Transcriptional analysis of spleen GC Tfh from untreated HIV+ subjects revealed a decrease in molecules involved in Tfh-B interaction such as CD40L, Ox40, and signaling lymphocytic activation molecule (SLAM) members, and reduced levels of IL-10 and IL-4 [93]. In contrast, GC Tfh showed higher levels of CXCL13 and IL-21 mRNA [93]. Another recent study reported the shift of GC Tfh towards an IL-21 single-producing phenotype with reduced polyfunctionality that correlated with increased plasma cell but decreased switched memory B cell frequencies [90], consistent with the role of IL-21 to promote plasma cell differentiation. Cubas et al. also detected a tendency for higher IL-21-production in GC Tfh from untreated HIV+ individuals [94]. However, the elevated expression of PD-L1 on GC B cells from triggered PD-1 signalling on co-cultured Tfh cells, which led to a reduced Tfh activation, proliferation and B cell help. In this study, addition of IL-21 to co-cultures rescued the impaired B cell help of LN Tfh cells from HIV+ donors [94].

Although the increase of Tfh cell frequencies in lymphoid tissue is not mirrored in peripheral blood of HIV-infected individuals, cTfh cells present qualitative defects in viremic and ART-treated subjects compared to uninfected controls, with a decreased ability to induce B cell differentiation and antibody production in vitro [95,96]. These results demonstrate phenotypic and functional changes of Tfh cells during HIV infection that contribute to impaired HIV-related and general B cell responses.

In untreated HIV infection, bNAbs typically develop after a few years of chronic antigen exposure. Because of immune escape of the autologous strain, these humoral responses are not associated with viral control [8]. These findings show that in a subset of HIV+ individuals, the immunological environment allows acquisition of the extensive hypermutations necessary for bNAb generation. Several groups subsequently investigated Tfh responses in subjects with good vs. poor neutralization to find correlates of efficient help. Studies in SHIV-infected rhesus macaques demonstrated a positive correlation between IL-4+ Env-specific LN Tfh and the frequency of Env-specific IgG+ GC B cells as well as neutralization breadth [97]. Further transcriptional analysis of these Env-specific Tfh cells revealed that animals with greater neutralization activity showed a higher expression of Tfh-related (Bcl6, MAF, CXCL13, and IL-21) and Th2-related genes (GATA3), whereas Th1- and Treg-related signatures (TBX21, IFNγ, or FoxP3) were reduced [97].

Correlates between Tfh responses and development of bNAbs in humans have been largely restricted to the analysis of blood as access to lymphoid tissue is limited. Differences in the cTfh response between HIV+ subjects with high or low levels of neutralizing antibody activity were detected with a high frequency of CXCR3-PD-1+ cTfh being associated with the development of bNAbs [67,98]. During the early phase of infection, individuals who later developed broad neutralization already showed superior Tfh and B cell responses compared to study participants who remained low neutralizers [99,100,101]. They demonstrated an enhanced ability of cTfh cells to induce antibody class-switching in vitro, a preserved B cell activation profile comparable to uninfected controls, and elevated levels of plasma CXCL13, which is a marker of GC activity. Furthermore, HIV-infected donors with bNAbs showed a higher level of plasma autoantibodies and less functional regulatory CD4+ T cells with elevated PD-1 expression, which limited their suppressive capacity [98]. Together, these results demonstrate that a high GC activity and Tfh quality seems to be important for the development of high-affinity antibody responses.

However, most studies mentioned rely on the analysis of the bulk Tfh population. Further studies are necessary to specifically investigate HIV-specific Tfh responses and the correlation of phenotype and function with protective antibody development, which might differ from observations made on total Tfh cells. Such investigations have been hampered by the limited tools available to study T cell antigen specificity independently of cytokine production. As Tfh show only limited cytokine production upon activation, they are more likely to be missed with standard assays such as intracellular cytokine staining or enzyme-linked immunospot (ELISPOT). Activation-induced marker (AIM) assays, recently developed by our group and others [102,103,104], overcome these limitations and allow the identification and study of antigen-specific Tfh responses in the tissue and blood during HIV infection and vaccination. These approaches, along with other tools such as Class II tetramers, will further help to decipher correlates of HIV-specific Tfh responses for the generation of protective antibody responses during HIV infection in humans, which will help guide strategies to induce such responses during vaccination.

## 7. Induction of Tfh Responses during Immunization

Classical vaccination strategies, while successful for the induction of protective antibodies against various pathogens, have failed to induce protective and long-lasting humoral responses against HIV. Given the importance of the quantity and quality of Tfh cells for the development of effective antibody responses in natural HIV infection several groups sought to determine how Tfh responses could be induced or modulated to improve humoral immunity (selected strategies are summarized in Table 3).

### 7.1. Use of Adjuvants to Direct Tfh Formation

Adjuvants are used to promote or shape immune responses during vaccination as the vaccine antigen alone is in most cases of low immunogenicity. They can enhance immune cell infiltration and antigen uptake into antigen-presenting cells (APCs) as well as activate innate immune cells via binding to specific receptors (e.g., toll-like receptor (TLR) agonists). Based on their different effects, specific adjuvants therefore guide distinctive types of generated immune responses. Alum is the most widely used adjuvant for vaccinations (>80% of all licensed vaccines) and has shown great success in vaccinations against for example hepatitis A and B, human papilloma virus, diphtheria, or tetanus [120]. However, alum alone induces low Tfh responses when compared to other adjuvants or when alum is combined with TLR agonists. A recently published study demonstrated that the oil-in-water adjuvant MF59 or a combination of alum and a TLR7 agonist promoted higher GC Tfh responses in the draining LN compared to alum alone using an NHP immunization model [105]. This was associated with an enhanced ability of MF59 or alum/TLR7 agonist to induce APC recruitment to the infection site and subsequent antigen uptake and presentation in the LN [105]. MF59 is approved as vaccine adjuvant in humans and did not only promote the generation of high titer antibody responses after flu immunization in children and elderly [121,122], who usually experience low efficacy, but increased the quality of antibody responses via enhancing cross-clade neutralization and affinity [123,124]. Total and influenza-specific ICOS+ cTfh were detectable in the blood of MF59-adjuvanted flu vaccine recipients and correlated with protective antibody titers [125].

Given the success of MF59 to enhance protective antibody titers in vaccines against other pathogens, it is currently used in some HIV vaccine efficacy trials, such as the HVTN702 study (ALVAC + gp120), conducted in South Africa (https://clinicaltrials.gov/ct2/show/NCT02968849?term=HVTN702&rank=1, accessed 27 May 2018). This study is based on the RV144 trial (ALVAC/AIDSVAX), which demonstrated partial antibody-mediated protection [126]. Compared to unsuccessful HIV vaccine trials, e.g., using a DNA/rAd5 vaccine regimen, vaccinees of the RV144 trial demonstrated a higher frequency of HIV-specific IL-21+ cTfh cells [127], which may suggest better Tfh help. This finding deserves further investigation and needs to be confirmed in other trials. Studies in NHPs mimicking the HVTN702 vaccine trial demonstrated a lower efficacy and protection against SIV acquisition despite a higher immunogenicity of MF59 when compared to alum [128]. Whether this will also be the case in the human trial remains to be determined and suggests, that a better understanding of adjuvant-mediated humoral responses is still required.

Other adjuvants that demonstrated a strong induction of GC Tfh responses include agonists for TLR4 [106], TLR7/TLR8 [107], and TLR9 [108] alone or in combination [109]. TLRs are either expressed on the surface or on intracellular compartments of APCs, B cells, and T cells and induce, when triggered, a pro-inflammatory immune response characterized by the expression of cytokines, chemokines, and co-stimulatory molecules [110]. Activation of TLRs of DCs induces the upregulation of molecules important for the interaction with naïve CD4+ T cells in the LN and, depending on the type of TLR activated, a specific cytokine and signalling molecule profile that can direct T cell differentiation [110]. For example, TLR3, TLR8, or TLR9 activation in APCs leads to the secretion of Tfh-promoting cytokines that mediated Tfh differentiation such as IL-6 in mice in vivo [111] or IL-12 in human APCs in vitro [107]. TLR9-signalling can also directly act on B cells and regulate affinity maturation and class switch [108]. In addition, immunization with TLR4 and TLR9 agonist adjuvants reduced the Tfr/Tfh ratio in the GC [108,112], which could lead to enhanced Tfh and B cell function. Immunomodulatory adjuvants for the induction of Tfh responses have to be carefully evaluated as Tfh-promoting cytokines differ between humans and mice (see Table 1). Therefore, given the close similarities between the NHP and human immune systems, NHPs are likely to be the best animal model for preclinical studies involving the modulation of Tfh responses. In a recent NHP study, immunization with the adjuvant Iscomatrix or using poly(lactic-co-glycolic acid) (PGLA)-nanoparticle encapsulated TLR4 and TLR7/8 agonists led to the induction of high frequencies of HIV-vaccine-antigen-specific GC Tfh responses, which correlated with antibody responses, when compared to alum [129]. In this study, GC Tfh responses could be followed longitudinally using fine needle aspiration (FNA) sampling of draining LNs. This technique involves inserting a needle, frequently under ultrasound guidance, that removes a small representative cell fraction of the total LN cell population [130]. FNA probing of LNs is well tolerated, minimally invasive and can serve as vaccine immune response monitoring system in future human vaccine trials.

In summary, these results demonstrate that multiple aspects of the GC responses can be modified by TLR agonists and their success in NHP studies suggest that they may also be beneficial for human vaccine development.

Given the importance of defined cytokines in promotion of Tfh responses, it is also possible that direct administration of these cytokines in combination with vaccination could enhance frequencies of GC Tfh cells as well.

### 7.2. Route of Vaccine Administration

Most vaccines are administered by intramuscular (i.m.) injection as this vaccination route demonstrated only minimal adverse effects [131]. However, subcutaneous (s.c.) or intradermal (i.d.) injection triggered an elevated differentiation of GC Tfh cells and increased antibody responses compared to i.m. vaccination in mice and NHPs [113,114]. This could be due to multiple factors. Soluble dye tracking in NHPs demonstrated superior drainage to local LNs after s.c. injection compared to the i.m. route [113].

Another factor is the differential distribution of DCs between tissue compartments. As mentioned before, DCs regulate CD4+ T cell differentiation. In a recent study, Krishnaswamy et al. identified migratory CD11b+ type 2 DCs as superior inducers of Tfh generation in mice due to their enhanced homing potential to the T-B border in secondary lymphoid organs [115]. These CD11b+ DCs and other APCs are located at high concentration in the skin but not in the muscle. In humans, i.d. immunization has shown better induction of antibody responses or similar responses using a lower dose upon vaccination against hepatitis B, influenza or human papilloma virus compared to i.m [131]. In a NHP study, GC Tfh frequency and HIV-neutralizing antibody titers were higher in animals that were immunized s.c. vs. i.m. [113]. In addition, animals were immunized s.c. at two different sites to ensure the engagement of more T and B cells into the GC. These results suggest that i.d. or s.c. injection could serve as favourable immunization route to enhance LN antigen delivery and increased systemic antibody responses in HIV vaccine studies. However, as mentioned before, i.m. and i.d. vaccination are associated with higher risks of adverse side effects compared to i.m. [131].

Since HIV is mainly transmitted across mucosal surfaces, an effective HIV vaccine likely depends on the generation of humoral responses in the mucosa. Mucosal antibody responses can be induced by systemic immunization routes, however, the exact mechanisms and how to specifically induce them via s.c., i.m., or i.d. immunization are not known [132]. Mucosal immunization routes (e.g., oral, intranasal, intratracheal, intrarectal, and intravaginal) have shown some success in NHP studies but have failed thus far in human HIV-vaccine trials (reviewed in [116]). Further studies will show whether mucosal vaccination alone or in combination with systemic immunization will serve as a strategy to induce protective immune responses against HIV in humans.

### 7.3. Enhanced or Extended Administration of Antigen to Induce and Maintain Tfh

As mentioned earlier, Tfh differentiation is promoted by prolonged DC-T interaction and Tfh maintenance is dependent on persistent antigen availability in the GC, suggesting that Tfh differentiation could be regulated by the amount of antigen given during vaccination. Indeed, GC Tfh and B cell responses are elevated when mice were immunized with higher doses of antigen [22]. Similarly, high dose immunization enhanced Tfh and antibody responses to influenza immunization in humans [133,134].

An alternative strategy to enhancing the dose of the single vaccine infections is the prolonged administration of antigen. Single injection of a vaccine antigen might not recapitulate the sustained availability of antigen in the GC during the natural infection of replicating pathogens. This might be especially important for the generation of HIV-specific bNAbs as they require extensive SHM acquired during multiple rounds of GC reactions [9]. To manipulate the kinetics of antigen availability, Tam et al. compared multiple or constant antigen delivery vs. single bolus injection during the prime immunization in mice [117]. Prolonged antigen exposure during the prime led to sustained antigen retention on FDCs in the LN, increased GC Tfh and B cell responses and elevated antigen-specific antibody titers in the plasma compared to single injection [117]. This strategy also showed success in HIV-Env-immunized NHPs: Animals that received the antigen continuously with osmotic pumps showed higher titers of tier 2 neutralizing antibody responses when compared to single injected animals [113].

Continuous antigen delivery can also be achieved using immunization with mRNAs encoding for the antigen of interest (reviewed in [135]). Single intradermal injection of an mRNA-containing immunization system encoding for HIV envelope, influenza hemagglutinin, or zika virus premembrane and envelope proteins led to protein expression for a prolonged time period and induced strong GC Tfh responses in immunized mice and NHPs [118]. Such de novo synthesis of immunogen in vivo-or extended release of immunogens shielded from protein degradation-may also be beneficial for preservation of conformational epitopes, for example for Env trimeric constructs.

## 8. Inhibiting Negative Regulators of GC Responses

Tfr cells represent an interesting target for inhibition to enhance GC Tfh and B cell function. Besides influencing Tfr/Tfh ratio at the differentiation level using certain TLR-agonists as mentioned before, Tfr cell function was directly targeted in mice. CTLA-4 blockade or knockout led to a decreased suppressive capacity of Tfr cells and augmented Tfh cell frequency and function, GC responses, and antigen-specific antibody responses upon immunization [50,119]. However, generated antigen-specific antibody responses were of lower affinity and sustained suppression of Tfr led to the emergence of auto-reactive antibodies [50,119]. Thus, Tfr cell-dependent suppression of GCs might actually be beneficial for the generation of effective antibody responses: only high-affinity B cell clones, which receive more activation signals, might be able to overcome Tfr-mediated suppression to proliferate and survive [85]. More studies are therefore necessary to understand the role of Tfr cells in regulating GC responses and the development of high-affinity antibody responses.

## 9. Future Directions

Current HIV-related vaccine studies in NHP animal models demonstrate that several new vaccination strategies alone or in combination are able to enhance GC Tfh and B cell responses, which will serve as basic concept for future studies. These strategies aim to enhance GC Tfh responses by broadly augmenting Tfh frequency, yet the question remains whether more targeted approaches and the induction of specifically efficient Tfh cells might be favourable for the generation of bNAbs upon vaccination. For this, a better understanding of Tfh function in the GC is indispensable.

This review focuses on the development of strategies for a prophylactic HIV vaccine. However, enhancing antiviral responses also plays a role in HIV strategies of a functional cure that aims at inducing control of any residual viral replication by the host’s own immune system in the absence of ART. Administration of bNAbs in ART-treated HIV+ subjects delayed viral rebound after analytic treatment interruption until viral resistance mutations occurred [136], suggesting that HIV-specific humoral responses might contribute to such control. A better understanding of quantitative and qualitative HIV-specific Tfh responses is necessary to evaluate whether active vaccination might serve as a strategy to induce more effective HIV-specific humoral responses in HIV+ individuals, or whether passive administration of bNAbs or long-acting antiretroviral drugs may be a more realistic approach.

## 10. Conclusions

Tfh responses have been shown to be involved in the generation of protective antibody responses in various infectious diseases and after vaccination. Induction of more potent Tfh responses represents an interesting strategy for vaccines that show limited efficacy, including HIV. Understanding Tfh biology has allowed developing alternative strategies that aim to induce more efficient Tfh responses, which have shown some success in NHP studies. However, further efforts are needed to determine whether these strategies can be successfully applied to HIV vaccine trials in humans.

## Figures and Tables

**Table 1:** Signalling molecules regulating Tfh differentiation in mice and humans.

Signalling Molecule/Receptor Pair	Species	Role in Tfh Differentiation	Source/Interacting Cell Type	References
**IL-6/IL-6R**	Mouse	Promotion	DCs, B cells	[24,25]
**IL-12/IL-12R**	Mouse, human	Promotion	DCs	[26,27,28]
**IL-21/Il-21R**	Mouse	Promotion	T cells	[29,30]
**IL-23/IL-23R**	Human	Promotion	DCs	[31]
**IL-27/IL-27R**	Mouse	Promotion	DCs	[32,33]
**IFN-γ/IFN-γR**	Mouse	Promotion	T cells	[34]
**TGF-β/TGF-βR**	Mouse, human	Inhibition (mouse), Promotion (human)	DCs	[31,35,36]
**Activin A/Activin-R**	Human	Promotion	DCs	[37]
**Ox40L/Ox40**	Mouse, human	Promotion	DCs, B cells	[20,38]
**ICOSL/ICOS**	Mouse, human	Promotion	B cells	[39,40,41]
**B7/CD28**	Mouse	Promotion	DCs, B cells	[42,43]
**SLAM family members**	Mouse, human	Promotion	B cells	[44,45]
**IL-2/IL-2R**	Mouse, human	Inhibition	T cells	[37,46,47,48]
**IL-7/IL-7R**	Mouse	Inhibition	DCs	[49]
**B7/CTLA-4**	Mouse	Inhibition	-	[42,50]

**Table 2:** Tfh mediators for B cell activation, differentiation and affinity maturation.

Tfh Functional Molecule	Effect on B Cells	References
**IL-21**	CSR, activation, proliferation, SHM, plasma cell differentiation	[57,58,59]
**IL-4**	Proliferation, CSR, SHM	[60,61]
**IL-10**	Proliferation, CSR, plasma cell differentiation	[62,63]
**CD40L**	Activation, proliferation, CSR	[64]

**Table 3:** Summary of strategies to enhance Tfh responses.

Vaccine Component	Strategy	Result	Effect on Tfh	Potential Caveat	References
**Adjuvant**	Alum + TLR agonists, MF59	Enhanced APC recruitment to infection site and elevated antigen delivery to LN	Tfh differentiation and maintenance	Increased immunogenicity might cause increased local and systemic adverse effects	[105]
	Various vaccine formulations containing TLR agonists	Induction of Tfh-promoting signals in DCs	Tfh differentiation		[106,107,108,109,110,111,112]
		Reduction of Tfr/Tfh ratio	Enhanced Tfh function		
**Route of vaccination**	Subcutaneous vs. intramuscular	Enhanced drainage of soluble antigen	Tfh differentiation and maintenance	Overabundant Tfh might lead to the selection of low affinity B cell clones (also applies to other strategies)	[113,114,115,116]
	Intradermal vs. intramuscular	Targeting of higher DC number	Tfh differentiation		
	Mucosal alone or in combination with systemic	Enhancing mucosal antibody responses	Direct site of humoral response		
**Enhanced or extended antigen delivery**	Increased antigen dose, prolonged antigen delivery using multiple injections, osmotic pumps or mRNA systems	Enhanced DC-naïve CD4 interaction that promotes Tfh differentiation, sustained availability of antigen on FDCs	Tfh differentiation and maintenance	Excessive long-term antigen persistence may be detrimental (exhaustion)	[22,113,117,118]
**Inhibition of negative regulators of Tfh**	CTLA-4 blockade	Limit suppressive function of Tfr, direct effect on Tfh	Enhanced Tfh function	Systemic blockade of immune checkpoints can have serious side effects-local blockade at site of delivery might be an alternative	[50,119]

TLR: toll-like receptor: APC: antigen-presenting cell; Tfr: follicular T regulatory cell; DC: dendritic cell.
